# An Interdigital Capacitor for Microwave Heating at 25 GHz and Wideband Dielectric Sensing of nL Volumes in Continuous Microfluidics

**DOI:** 10.3390/s19030715

**Published:** 2019-02-10

**Authors:** Tomislav Markovic, Juncheng Bao, Gertjan Maenhout, Ilja Ocket, Bart Nauwelaers

**Affiliations:** 1 Division ESAT-TELEMIC, KU Leuven, Kasteelpark Arenberg 10 box 2444, 3001 Leuven, Belgium; Juncheng.Bao@kuleuven.be (J.B.); Gertjan.Maenhout@kuleuven.be (G.M.); Ilja.Ocket@imec.be (I.O.); Bart.Nauwelaers@kuleuven.be (B.N.); 2 imec, imec PERSYBE Group, Kapeldreef 75, 3001 Heverlee, Belgium

**Keywords:** continuous microfluidics, dielectric sensing, microwave heating, interdigital capacitor

## Abstract

This paper proposes a miniature microwave-microfluidic chip based on continuous microfluidics and a miniature interdigital capacitor (IDC). The novel chip consists of three individually accessible heaters, three platinum temperature sensors and two liquid cooling and mixing zones. The IDC is designed to achieve localized, fast and uniform heating of nanoliter volumes flowing through the microfluidic channel. The heating performance of the IDC located on the novel chip was evaluated using a fluorescent dye (Rhodamine B) diluted in demineralized water on a novel microwave-optical-fluidic (MOF) measurement setup. The MOF setup allows simultaneous microwave excitation of the IDC by means of a custom-made printed circuit board (connected to microwave equipment) placed in a top stage of a microscope, manipulation of liquid flowing through the channel located over the IDC with a pump and optical inspection of the same liquid flowing over the IDC using a fast camera, a light source and the microscope. The designed IDC brings a liquid volume of around 1.2 nL from room temperature to 100 °C in 21 ms with 1.58 W at 25 GHz. Next to the heating capability, the designed IDC can dielectrically sense the flowing liquid. Liquid sensing was evaluated on different concentration of water-isopropanol mixtures, and a reflection coefficient magnitude change of 6 dB was recorded around 8.1 GHz, while the minimum of the reflection coefficient magnitude shifted in the same frequency range for 60 MHz.

## 1. Introduction

Point-of-care diagnostics is a novel front to fight disease in non-laboratory settings [1]. A smart design of cross-disciplinary devices, requiring minimal technical knowledge for device handling, brings new possibilities closer than ever to diverse healthcare environments [1]. These new possibilities are viable due to invested efforts in development and integration of sensor, actuation and microfluidics technologies. Sensing technologies in the microfluidic environment are numerous [2,3,4,5,6,7,8,9,10] and most often present optical [7,8] or electrical [3,4] signal at the output of a measurement transducer. In particular, every sensing technology has its advantages and disadvantages, with respect to price, size, integration capability, multi-functionality, need for labels, fabrication methods, and many other [10,11]. Thus many parameters [10,11], such as sensitivity, specificity, selectivity, minimal sensing volume, nonlinearity, detection limit, time characteristics, power requirements, and hysteresis of a sensor, exist in practice to compare and select sensing devices to match required needs. In this work, electrical sensing devices are investigated because they offer one very distinctive advantage—the useful dual role of both dielectric sensing and thermal excitation once employed at microwave and mm-wave frequencies. Additionally, when these electrical devices having the dual role are fused with nanoliter volume samples in microfluidics, the newly obtained combination becomes an interesting option to tackle challenges in diagnostics due to many desired operational characteristics. Some of these characteristics are rapid thermal actuation, label-free sensing and minimal consumption of samples. 

Several authors have demonstrated microwave-microfluidic chips for heating of nanoliter droplets flowing through a fluidic channel [12,13,14,15,16,17,18]. For that purpose, microwave transmission lines [12,13,14,15], planar split ring resonators [16,17] and coupled lines [18] were employed. In addition to heating, planar split ring resonators were used to demonstrate the dual capability of microwave devices. Boybat et al. [18] demonstrated microwave heating on 2 nL droplets having a residence time of 5.62 ms in the heating region, during which their temperature was increased by 42 °C using 27 dBm at 3 GHz. The same resonator demonstrated dielectric sensing capability on different concentrations of a water-glycerol mixture. Abduljabar et al. [17] employed a double split ring resonator as well for microwave heating of liquid in a fluidic channel at 2.86 GHz. The change of the resonant frequency was used for temperature measurements, proving the dielectric sensing capability of the developed electrical device. In addition to microwave device for both heating and sensing of nanoliter droplets in continuous microfluidics, several authors have reported work on dielectric sensing of liquids in continuous microfluidic platforms [19,20,21,22,23,24,25,26]. Interdigital capacitors [19,20], a spiral-shaped microwave resonator [21], complementary split ring resonators [22,26], substrate integrated waveguides [23,25], a quarter wavelength resonator [24] and a dual-mode resonator [5] were used to measure biological aqueous solutions, different alcohols, aqueous solutions with different glucose concentrations and chemical solutions. In conclusion, the reported devices demonstrated both microwave heating and sensing, and possibilities of microwave dielectric sensing in droplet and single phase continuous microfluidics. Nevertheless, the reported heating devices require an area on a microfluidic chip that is proportional to the operating wavelength, which tends to be large at low microwave frequencies. Due to that reason, integration of multiple resonant-based microwave devices on a small microfluidic chip is not fully feasible. In addition to the required area to accommodate microwave resonators, presented devices most often operate at frequencies around 3 GHz. At these frequencies, conversion of microwave energy into heat is less efficient than at higher microwave frequencies from the perspective of complex permittivity of deionized water [27], because the imaginary part of the complex permittivity of water is smaller at 3 GHz in contrast to the peak values of the imaginary part at 25 GHz [27]. Moreover, the imaginary part is being reduced as temperature of water is being increased [27] and therefore less power is dissipated in heated liquid as temperature increases.

In this work, a miniature uniplanar microwave device for thermal actuation of nanoliter volumes in continuous microfluidic (CMF) channels, which also allows microwave dielectric sensing of liquids as previously reported work [19,20,21,22,23,24,25,26] is presented. The developed microwave device is built around the interdigital capacitor (IDC) topology that allows efficient and to a certain extent uniform heating at 25 GHz. Apart from heating, the IDC can be used for dielectric sensing of liquid in the channel once being excited by a low-power microwave signal. CMF channels are realized on a quartz substrate with poly (dimethylsiloxane) (PDMS), while the uniplanar IDC is realized in a gold metal layer on a quartz substrate. Next to the miniature IDC, this article presents a novel microwave-microfluidic chip containing three individually accessible IDCs and a fluidic design offering liquid mixing and cooling. The designed microwave-microfluidic chip is connected to an in-house custom-made microwave printed circuit board (PCB) using elastomer conductive pins, which allow electrical access to microwave devices on the fluidic chip. Fluidic access to the microfluidic channel on the developed chip is achieved using hollow magnets and standard tubing [28]. Additionally, this paper presents a MOF setup that allows simultaneous microwave excitation of IDCs on a novel-microwave-microfluidic chip, manipulation of liquid located over IDCs with a pump and optical inspection of the same liquid flowing over the IDC using a fast camera, a light source and the microscope. Finally, this article presents evaluation of heating and sensing performances of the designed IDC on the microwave-microfluidic chip using a temperature sensitive fluorescent dye (Rhodamine B) and different concentrations of a water-isopropanol mixture using the MOF measurement setup and the in-house custom made PCB. 

## 2. Microwave-Microfluidic Platform

### 2.1. Interdigital Capacitor Design

The microwave device is specified to sense and locally heat a nanoliter volume at a specific location in the fluidic channel on a microwave-microfluidic chip, without thermal actuation of liquid located at different points on the fluidic chip. Therefore, the heater realization in a close contact with liquid is needed. In addition, the heating device must be realized as a uniplanar structure to maintain optical access to the flowing liquid from one side of the fluidic chip. A feasible option for such a device is the interdigital capacitor (IDC) topology, which can locally create strong electric fields required for fast microwave dielectric heating. A split ring resonator is not chosen to realize the microwave device to comply with a small footprint requirement for a microwave device and a cavity resonator is not chosen due to the optical inspection requirement. Furthermore, given the optical inspection requirement, a co-planar waveguide (CPW) transmission line is chosen to feed the microwave heater instead of a microstrip transmission line. Aside from localized microwave heating, it is desired to heat nanoliter volumes in as uniform as possible fashion. Although non-uniform temperature profiles create internal vortices that mix nanoliter volumes fast and bring liquid to uniform temperatures after a certain amount of time, results of temperature-sensitive biological reactions are more accurate once heating itself is more uniform and the mixing time is shorter.

A small size of a heated volume (nanoliter range) in a small fluidic channel reflects in a small footprint of an IDC, which means that the self-resonant frequency of the IDC itself is very high. Consequently, the high self-resonant frequency of the IDC allows the operation of the IDC heater at high frequencies that brings high heating efficiencies in contrast to low IDC heating efficiency at low frequencies. Although the operation at very high frequencies is beneficial from the theoretical perspective of dielectric losses in deionized water, it is decided to design heaters at 25 GHz. By choosing so, the peak of the imaginary part of water complex permittivity [27] is exploited and the self-resonance of the IDC is avoided. Finally, the choice of the operating frequency of 25 GHz results in a small signal wavelength, which reflects in miniature supporting microwave components that do not require a lot of space.

**Figure 1:**
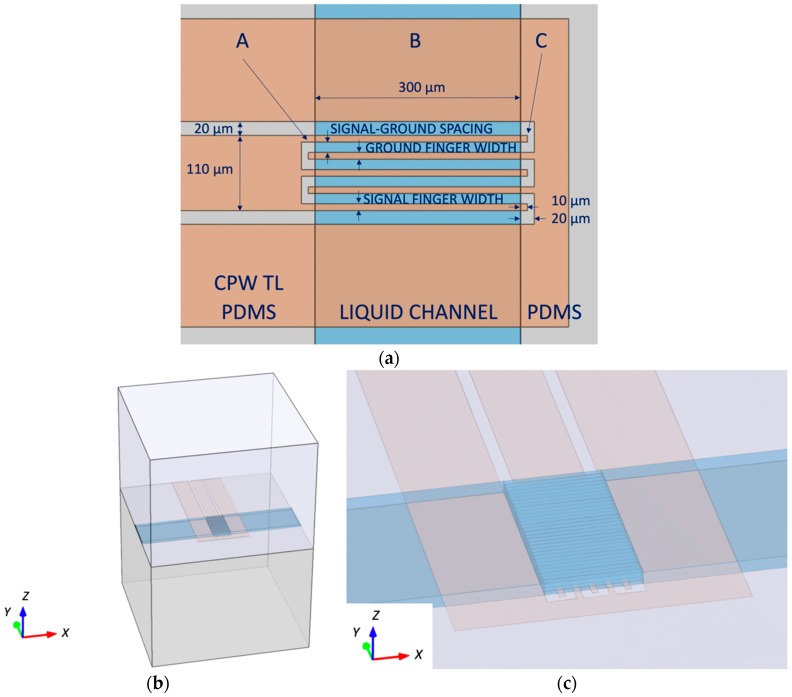
(**a**) The IDC design space: signal and ground finger widths and their mutual spacing. (**b**) A COMSOL Multiphysics^®^ simulation model of an IDC on quartz covered by PDMS; (**c**) A close-up view of 10 µm wide water sections used for optimizing IDC dimensions for uniform heating.

The layout of the investigated IDC topology for the desired nanoliter volume is split into three parts (A, B, C) that have different electric field distributions as indicated in Figure 1a. For heating applications, the most interesting is the middle part of the IDC that can be designed to have uniform transversal electric field distribution in signal-ground gaps. The beginning and the end of the IDC are not suitable for dielectric heating as they impose large electric field gradients, which heat liquid in the channel in a non-uniform manner. Therefore, PDMS channel walls are covering these parts of the IDC having large field gradients, while the remaining part of the IDC is exposed to the flowing liquid as indicated in Figure 1a. The design space of the investigated IDC heater consists of the signal and ground finger widths and their mutual spacing, as indicated in Figure 1a. To evaluate the heating performance of the designed device, a water volume located over the heater area is sliced into 10 µm wide sections as shown in Figure 1b,c, in which power losses are integrated and mutually compared. The design goal is to obtain an IDC design having standard deviation of integrated power losses smaller than 5% of the average value of integrated power losses. Therefore, based on values of integrated power losses in water sections, the IDC signal and ground width, and their mutual spacing is tuned to minimize standard deviation of dielectric losses in liquid. This layout optimization process is iterated until the design goal is achieved. The overall heating efficiency of the IDC is not optimized in this design case as the main focus is put on the heating uniformity—the IDC efficiency in heating can be later improved by means of a matching network. In addition to monitoring integrated power losses, foreseen heating uniformity is also evaluated by monitoring uniformity of the electric field in the liquid volume over the IDC during the layout optimization. The electric field profile is monitored in the heater transversal plane (*XY* plane) and across the channel height plane (*XZ* plane)–coordinate axes presented in Figure 1b. The field monitoring in the *XY* plane helps to take care of gradients occurring in the transversal electric field profile, while the field monitoring in the *XZ* plane deals with gradients in the vertical field profile.

**Figure 2:**
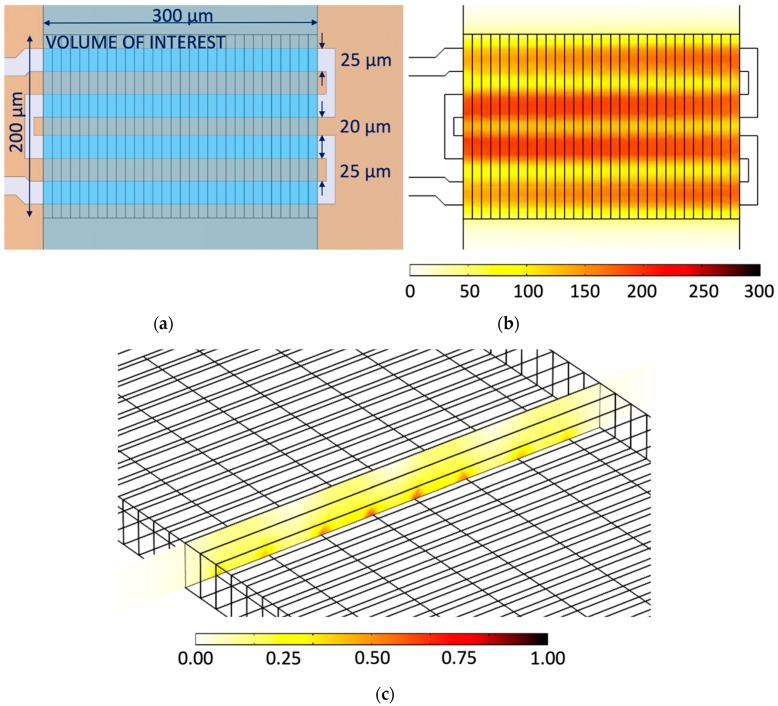
(**a**) The final layout of the IDC heater obtained in COMSOL simulations; (**b**) The electric field in kV/m in the middle of the channel in the *XY* plane—10 µm above the heater; (**c**) The electric field in MV/m in the middle of the fluidic channel in the *XZ* plane.

Electromagnetic (EM) simulations in the COMSOL Multiphysics^®^ software package were carried out on the device model presented in Figure 1b. The thickness of the quartz substrate (ε = 3.75 − j × 0.0015) and the PDMS (ε = 2.68 − j × 0.054) cover is set to 1 mm. Furthermore, dimensions of the substrate base are chosen to be 1.4 mm × 1.4 mm. These model dimensions are sufficient to capture the electromagnetic behavior of the IDC at the minimal computational cost. Metal layers are modelled as perfect electric conductors to avoid detailed mesh and computationally demanding EM design. The IDC heater for a water (complex permittivity values taken from [27]) volume of around 1.2 nL located over the heater is designed on the B section area of 300 µm × 200 µm (width × length) presented in Figure 2a. The channel, and consequently the liquid height is 20 µm in this design investigation. The final design dimensions of the IDC heater at 25 GHz for a volume of 1.2 nL using the previously described design methodology are found to be 25 µm for the signal-ground conductor spacing, 25 µm for the signal conductor widths and 20 µm for the ground conductor width as indicated in Figure 2a. The heater design also deals with the size of overlapping IDC regions located under PDMS channel walls—sections A and C indicated in Figure 1a. It is needed to maintain the length of these sections as short as possible to reduce their contribution on electric field distribution of designed devices. In other words, longer overlapping sections mean longer IDC devices, which at some point become comparable to a part of the operating wavelength. Once the size of devices becomes comparable to the operating signal wavelength, a standing wave pattern can exist, which naturally brings undesired gradients in electric field distribution. Therefore, the additional length of fingers and gap spacing in sections A and C is chosen to be 10 µm, which adds only 40 µm to the length of the IDC. Finally, the CPW transmission line dimensions are chosen to be 110 µm for the signal conductor width, 20 µm for the signal-ground spacing and 150 µm for the ground conductor width. These dimensions offer smooth transition between the transmission line and the IDC, and a transmission line characteristic impedance close to 50 Ohm—it is 57 Ohm for designed transmission line dimensions.

**Figure 3:**
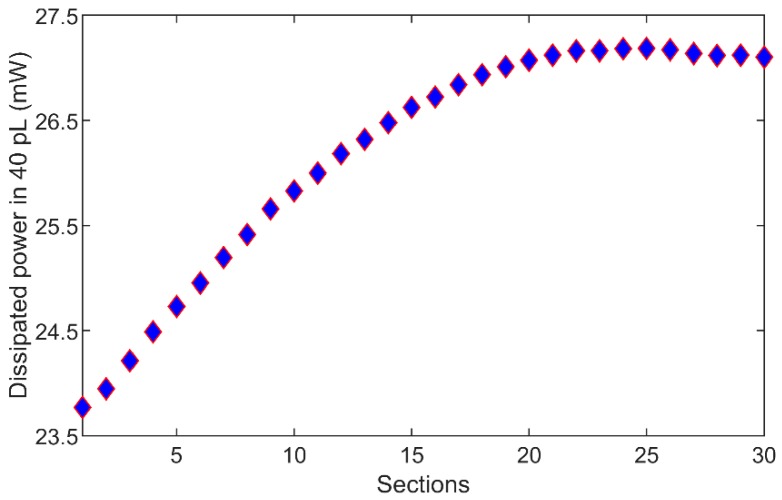
Dissipated microwave power in 30 water sections having a volume of 40 pL. Average dissipated power in 40 pL volumes is 26.19 mW for 1 W of input power, while standard deviation is 1.11 mW, which is 4.23% of the average value.

Electric field distribution of the designed IDC in the middle of the channel (10 µm above the heater) in the *XY* plane is presented in Figure 2b, while electric field distribution in the middle of the channel in the *XZ* plane is presented in Figure 2c. The *XY* average value of the electric field in the *XY* plane in Figure 2b is 134.66 kV/m with standard deviation of 3.83 kV/m, which is 2.84% of the *XY* average value. This indicates that the electric field in *XY* plane in the middle of the channel is relatively uniform. The *XZ* average value of the electric field in Figure 2c is 154.96 kV/m with standard deviation of 37.16 kV/m, which is 23.98% of the *XZ* average value. In other words, Figure 2c shows a stronger electric field close to the IDC electrodes and a weaker electric field more away from the electrode, both as expected. In the end, the maximum value of the electric filed in 30 water sections is 1.38 MV/m, while the minimum value is 10.13 kV/m. Power distribution along 30 water sections in direction of the IDC heater is presented in Figure 3. Average dissipated power in 40 pL volumes is 26.19 mW for 1 W of input power, while standard deviation is 1.11 mW, which is 4.23% of the average value—this means the design goal is met. Finally, the simulation model consisting of the IDC and a 0.7 mm long feeding transmission line dissipates 78.5% of input power in 30 water sections, 0.01% in quartz substrate, 0.3% in PDMS covering the feeding transmission line and 0.5% in water surrounding the 30 water sections in the channel.

### 2.2. Microwave-Microfluidic Chip Design

**Figure 4:**
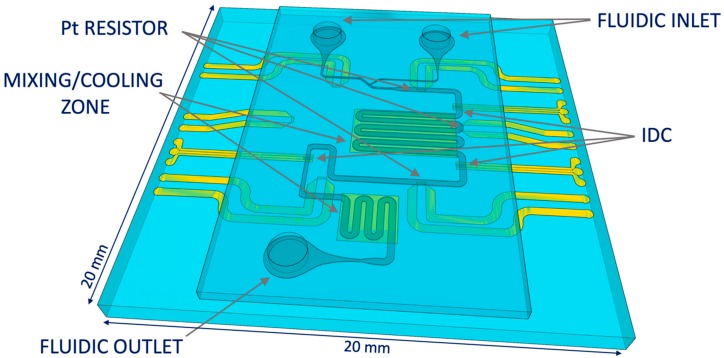
The microwave-microfluidic chip with three IDC microwave heaters, three platinum temperature sensors, two cooling pads and a fluidic channel with a T-junction.

Continuous microfluidic (CMF) channels are realized on a quartz substrate in poly (dimethylsiloxane) (PDMS). The use of PDMS allows fast prototyping of fluidic devices and optical inspection of investigated droplets. The fluidic chip design deals with design of fluidic inlets and outlets, channel dimensions and a fluidic T-junction used for droplet generation. In addition to the channel sizing, attention is also given to heat transfer occurring during droplet cooling. Fast cooling and liquid mixing are achieved by the meandered fluidic channel with metal pads underneath, used to diffuse heat to the bulk substrate. Individually addressable designed microwave heaters are placed on three locations around the chip to allow simultaneous heating and sensing. The microwave-microfluidic chip having fast microwave heating and enhanced cooling is presented in Figure 4. In addition, platinum resistors are designed for accurate local contact-based temperature measurements with a digital multimeter and minimal self-heating that does not influence temperature measurements of the flowing liquid. These platinum sensors are placed at three locations to measure temperature in between heaters as presented in Figure 4.

### 2.3. Microwave-Microfluidic Platform Design

**Figure 5:**
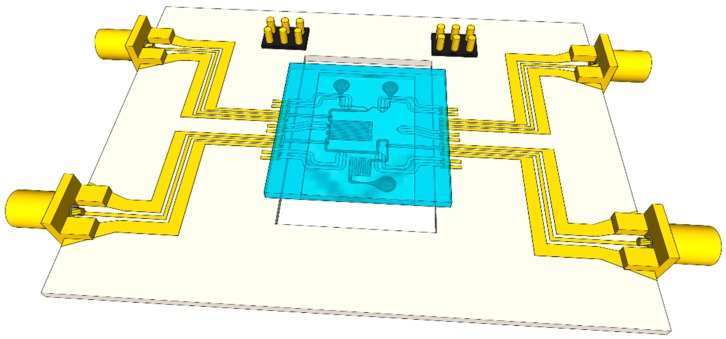
Illustration of the microwave-microfluidic platform with the microwave-microwave chip.

The microwave-microfluidic chip is connected using elastomer conductive pins to a custom-made microwave printed circuit board (PCB), which allows electrical access to heaters and Platinum resistors for local temperature sensing, as presented in Figure 5. Next to electrical access to the chip, the integrated microwave-microfluidic platform allows simultaneous fluidic actuation and optical inspection of liquid in the channel through an opening in the PCB. The microwave-microfluidic chip is manually positioned on the PCB, and pressed firmly to the PCB from the top, having a good electrical contact, using a spring-loaded plastic pin.

## 3. Experimental Results

### 3.1. Microwave-Optical-Fluidic Measurement Setup

**Figure 6:**
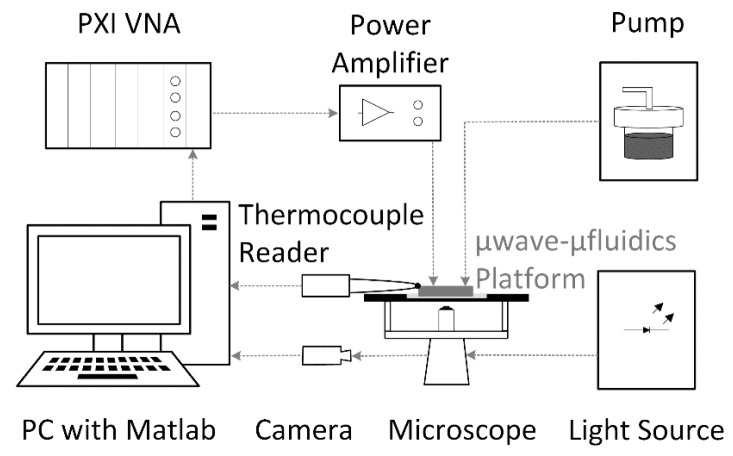
The block schematic of the microwave-optical-fluidic measurement setup.

Heating measurements are done on the microwave-optical-CMF measurement setup. The microwave part consists of a PXI M9735A vector network analyzer (VNA) (Keysight, Santa Rosa, CA, USA) and an 18–26.5 GHz microwave power amplifier (Fairview Microwave, Lewisville, TX, USA). The optical part consists of an IX73 microscope (Olympus, Tokyo, Japan), a pE-4000 light source (CoolLED, Andover, UK), an ORCA-Flash4.0 LT camera (Hamamatsu Photonics, Hamamatsu City, Japan), and ET548/10X and 59004m optical filters (Chroma, Bellows Falls, VT, USA). The microfluidic part consists of a MFCS-EZ pump (Fluigent, Paris, France) and tubes with magnets used to for the channel-tube sealing. The network analyzer is used as a microwave source exciting the microwave amplifier in heating tests. The optical part of the setup is used to measure the changes of the fluorescence intensity of the water-Rhodamine B mixture during the heating process. The change of the fluorescence is linked to the change of the liquid temperature once the base temperature of the liquid prior to the heating is known [29]—the temperature-sensitive fluorophore (Rhodamine B) is dissolved in water at a concentration of 1 mmol/L. So, when illuminated by the light source at 550 nm, the intensity of emitted light at about 625 nm changes as a function of temperature. The base temperature prior to heating measurements is measured with a TC 01 temperature logger from National Instruments (Austin, TX, USA) and a K-type thermocouple (TC). The complete measurement setup is controlled by the in-house developed Matlab code. The block schematic of the measurement setup is presented in Figure 6.

### 3.2. Microwave Heating Experiments

**Figure 7:**
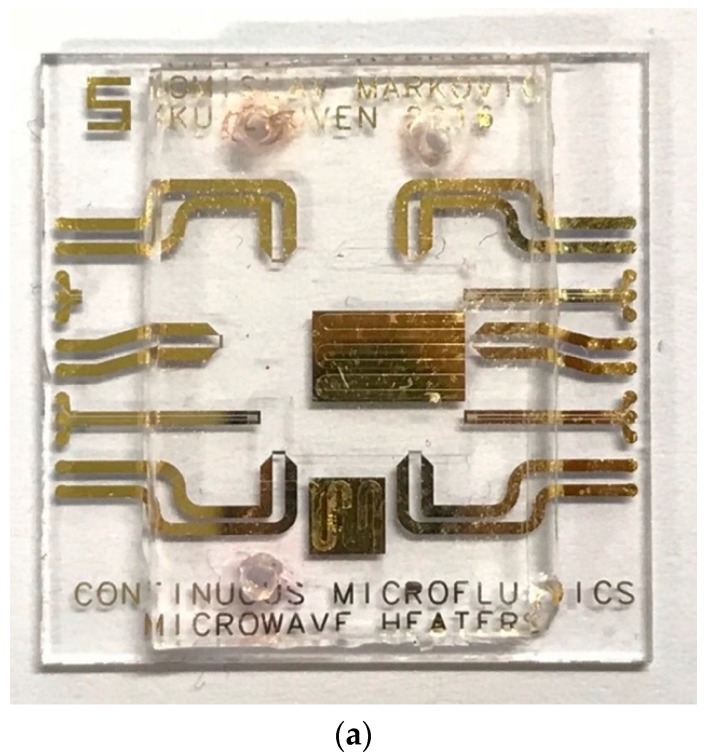

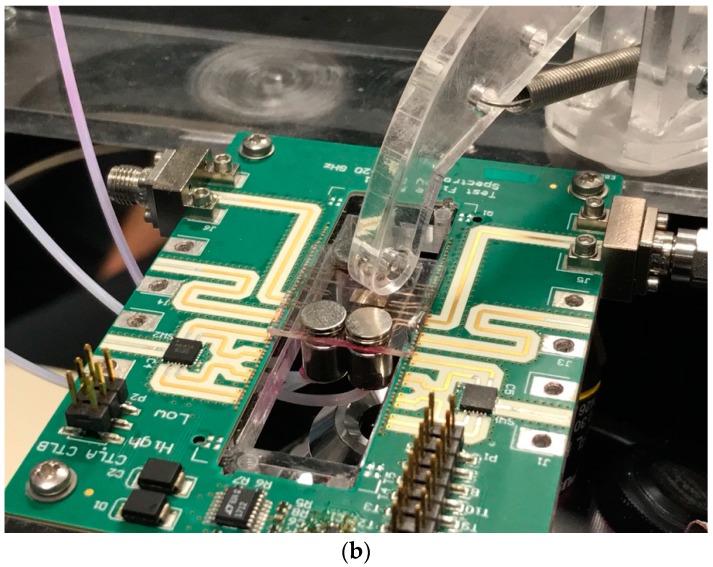
(**a**) The manufactured microwave-microfluidic chip (20 mm × 20 mm); (**b**) The microwave-microfluidic chip placed on top of the microwave-microfluidic platform and fixed by the spring-loaded plastic pin.

**Figure 8:**
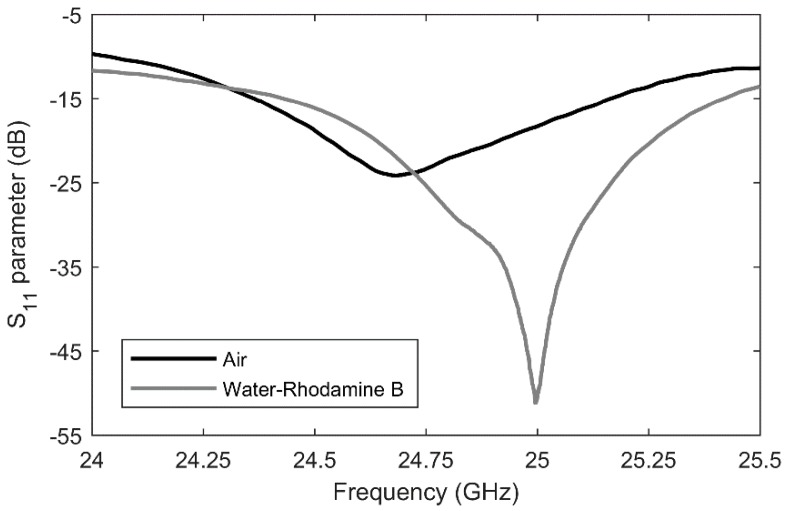
The S_11_ parameter at the coaxial connector reference plane on the feeding PCB loaded with the microwave-microfluidic chip in which the IDC heater is loaded with air and the liquid mixture at 23.5 °C.

A manufactured microwave-microfluidics chip with microwave heaters is presented in Figure 7a. Furthermore, Figure 7b shows the complete assembly of the microwave-microfluidics chip and the microwave-microfluidics platform, placed on a microscope top stage. Prior to heating measurements of a water-Rhodamine B mixture in concentration of 1 mmol/L, S_11_ parameter (a ratio of reflected and incident wave at the input port) of the heater with air and liquid mixture filled channel are measured. The chip is placed on the microwave-microfluidic platform in Figure 7b and measured using the PXI M9735A VNA. Measurement results at 23.5 °C are shown in Figure 8. Based on these measurement results, we can see that microwave power is being accepted at the coaxial connector reference plane on the PCB.

Heating measurements of the continuous stream of the water-Rhodamine B liquid mixture flowing through the microwave-microfluidic chip with microwave heaters for 1.2 nL volumes are done for four different cases:

(1) 0 mbar pressure difference between inlet and outlet,
(2) 100 mbar pressure difference between inlet and outlet,
(3) 200 mbar pressure difference between inlet and outlet,
(4) 300 mbar pressure difference between inlet and outlet.

**Figure 9:**
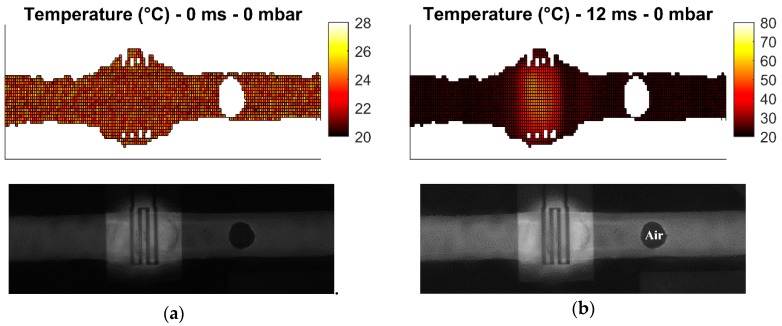

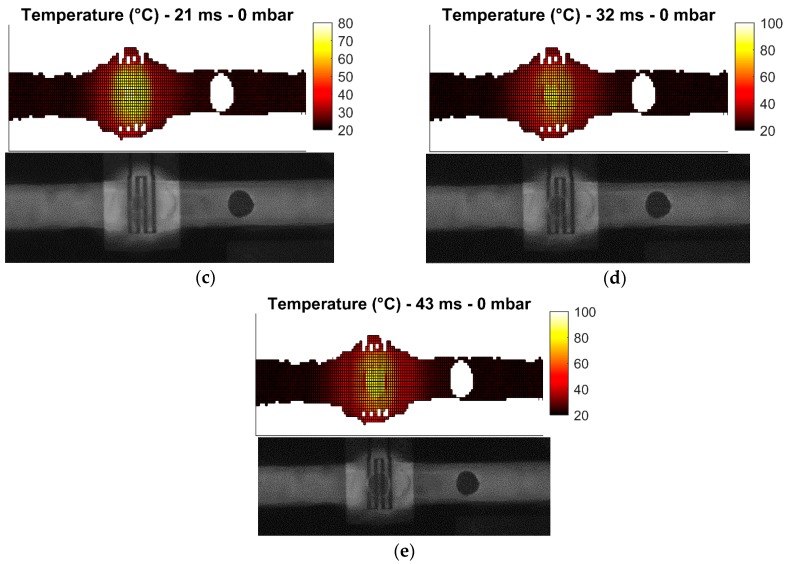
Measured temperature profile (top) of the fluorescent mixture in the channel with the corresponding image (bottom) from the camera for the pressure difference of 0 mbar after: (**a**) prior to heating; (**b**) 12 ms heating started; (**c**) 21 ms heating started; (**d**) 32 ms heating started; (**e**) 43 ms heating started.

**Figure 10:**
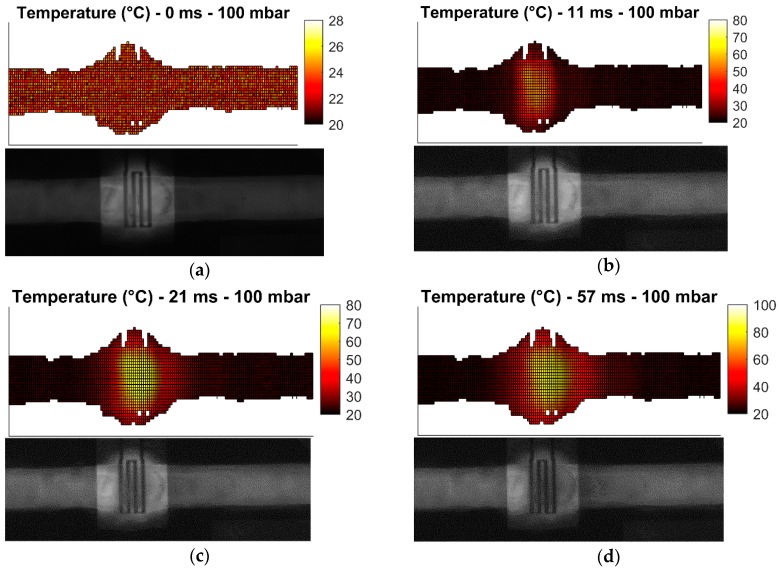

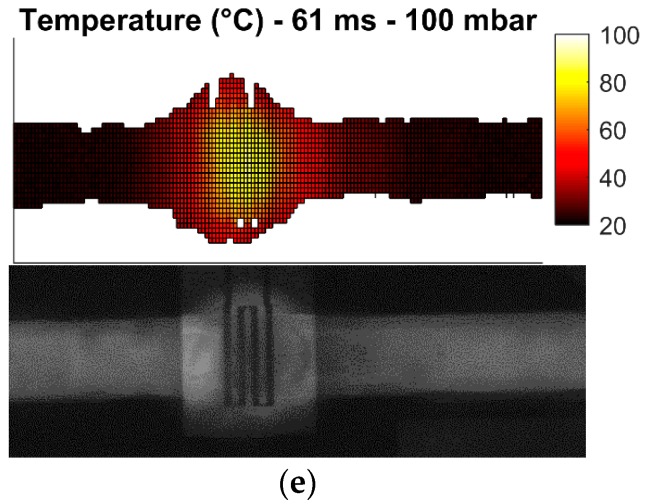
Measured temperature profile (top) of the fluorescent mixture in the channel with the corresponding image (bottom) from the camera for the pressure difference of 100 mbar after: (**a**) prior to heating; (**b**) 11 ms heating started; (**c**) 21 ms heating started; (**d**) 57 ms heating started; (**e**) 61 ms heating started.

**Figure 11:**
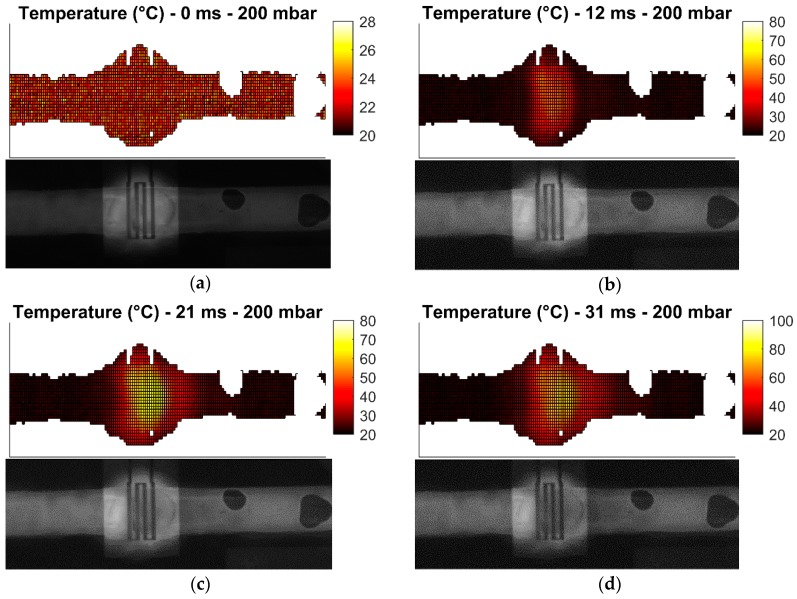

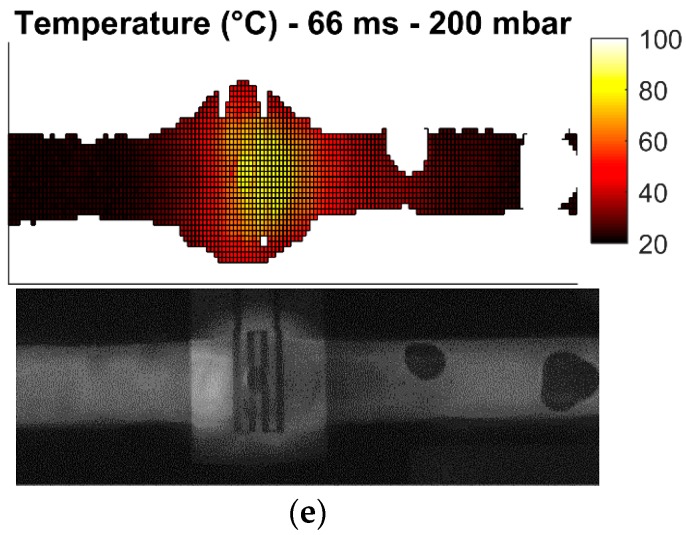
Measured temperature profile (top) of the fluorescent mixture in the channel with the corresponding image (bottom) from the camera for the pressure difference of 200 mbar after: (**a**) prior to heating; (**b**) 12 ms heating started; (**c**) 21 ms heating started; (**d**) 31 ms heating started; (**e**) 66 ms heating started.

**Figure 12:**
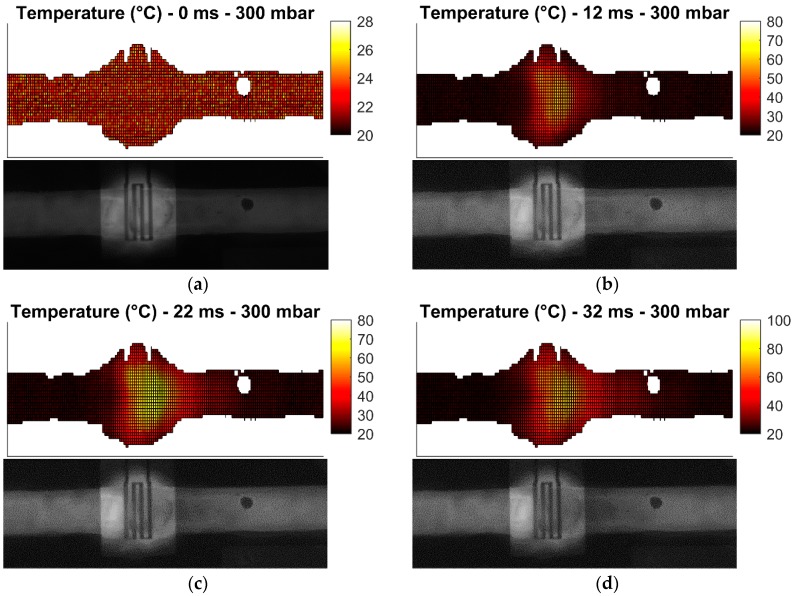

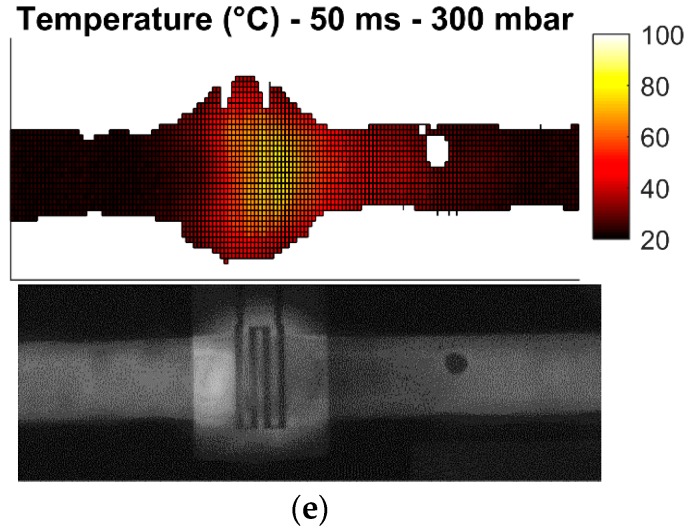
Measured temperature profile (top) of the fluorescent mixture in the channel with the corresponding image (bottom) from the camera for the pressure difference of 300 mbar after: (**a**) prior to heating; (**b**) 12 ms heating started; (**c**) 22 ms heating started; (**d**) 32 ms heating started; (**e**) 50 ms heating started.

These measurement cases allow the investigation of the heater performance in static and dynamic conditions. Heating of droplets is not investigated due to challenges in droplet generation. All measurements are carried out with 31.8 dBm (1.58 W) of power at 25 GHz at the input of the microwave-microfluidic platform. Losses of the transmission line and elastomer pins on the PCB, which are losses between the microwave-microfluidic chip and the output port of the amplifier, are not considered in heating measurements. Finally, images of the fluorescent mixture in the microfluidic channel are taken from the camera with the average time step of 10 ms. Measurement results are presented in Figure 9, Figure 10, Figure 11 and Figure 12.

Heating measurements demonstrate the capability of the microwave heater to bring the liquid stream in the channel almost instantaneously to 100 °C. More precisely, the IDC heats liquid above the heater area to 100 °C in 21 ms. As expected, it is the easiest to achieve this high temperature when liquid is not flowing, as presented in Figure 9b,c. Once liquid starts flowing, the amount of time to transfer electric energy into heat in liquid is reduced, which consequently results in lower achieved temperatures. In addition, the heated liquid is pushed down the stream where it interacts with the cooler liquid and losses generated heat to the cool liquid and environment—when liquid is not flowing, heat is built up in the immediate environment around the heater and consequently heat losses from the heated liquid to the passively heated environment are lower due to the smaller temperature difference. This effect can be seen by comparing data in Figure 9 with data presented in Figure 10. Furthermore, once the speed of the flowing liquid is increased by a larger pressure difference between the fluid inlet and outlet, the reduced interaction time and the cool liquid and environment effect become more pronounced, as can be seen by comparing data in Figure 10 with data in Figure 11. Finally, once liquid is flowing relatively fast in the channel, the maximum achieved temperature is noticeably lower than the maximum temperature of the liquid stream that is not moving—this can be seen by comparing data in Figure 9 with data in Figure 12. Although the effect of fast flowing speed is present during the liquid stream heating with microwaves, it is interesting to observe and compare heating rates at the beginning of heating for all measurement cases. Namely, we are considering data in Figure 9b, Figure 10b, Figure 11b and Figure 12b. In all these Figures, heating rates are very similar, which means the microwave heater is performing well at the very beginning of heating. This means microwave heat generation is fast in the beginning of heating. Later, cool liquid comes to the heater and mixes with the heated part of the volume as previously described, finally influencing the temperature profile of the liquid before and after the heater zone. Additionally, due to the local change of the liquid temperature, the imaginary part of the complex permittivity is changed and the liquid heating process is locally altered.

In summary, the complete heating process is reduced to the fact that heat generated by the microwave heater in fast flowing streams is being spread over a channel length, forming a comet-tail alike temperature profile. Although the elongated temperature profile exists in fast flowing liquids, heating is still localized and limited only to the heater area as desired prior to investigating microwave heaters—this is confirmed by the localized temperature increase of liquid in Figure 9, Figure 10, Figure 11 and Figure 12 and electric field plots of heaters in Figure 2.

### 3.3. Microwave Sensing Experiments

**Figure 13:**
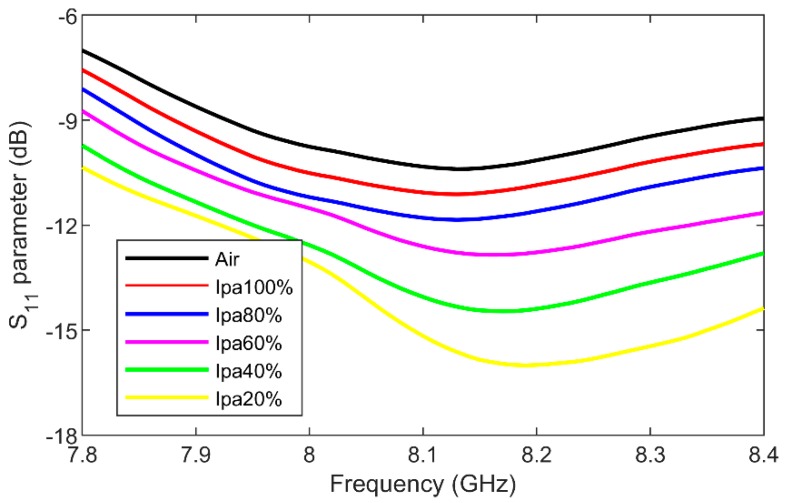
The S_11_ parameter of the combined custom-made PCB with loaded microwave-microfluidic chip filled with different concentrations of the water-isopropanol mixture.

The designed IDC for thermal actuation of the liquid located in the fluidic channel is also employed for dielectric sensing in the microwave frequency range. Due to the fact that the impedance of the IDC is dependent to the complex permittivity of the loaded liquid, different concentrations of a water-isopropanol (IPA) mixture were flushed through the microfluidic channel on the microwave-microfluidic chip and reflection coefficient measurements were taken at the coaxial connector mounted on the custom-made PCB presented in Figure 7b. These different impedances of the IDC further change the input impedance at the coaxial connector reference plane on the PCB. Measurements were carried out in a frequency range from 7.8 GHz to 8.4 GHz due to the valley of reflection coefficient magnitude of the combined novel microwave chip and the custom-made PCB. Reflection coefficient measurement results for different concentrations of the loaded mixture are presented in Figure 13. A clear difference between all liquid samples can be observed in a wide frequency range for both the minimal magnitude value and corresponding frequency point of the reflection coefficient. A reflection coefficient magnitude change of 6 dB was recorded around 8.1 GHz, while the minimum of the reflection coefficient magnitude shifted in the same frequency range for 60 MHz.

## 4. Conclusions

In this paper, an IDC-based microwave heater and sensor realized on a quartz substrate for continuous microfluidics realized in poly(dimethylsiloxane) was designed. The miniature IDC device was used to conceive a novel microwave-microfluidic chip offering electrical and fluidic functionality. The novel chip consists of three individually accessible heaters, three Platinum temperature sensors and two liquid cooling and mixing zones. The manufactured microwave-microfluidic chip with heaters for nL volumes was evaluated using the novel microwave-optical-microfluidics measurement setup. The microwave heater located on the chip is measured for several different flow speeds achieved using a pressure difference of 0 mbar, 100 mbar, 200 mbar and 300 mbar between the fluidic inlet and outlet. The manufactured heater brings still liquid stream above the heater area to 100 °C in 21 ms. Dielectric sensing capabilities of designed and manufactured IDC device were evaluated on the water-isopropanol mixture in different concentrations. A reflection coefficient magnitude change of 6 dB was recorded around 8.1 GHz, while the minimum of the reflection coefficient magnitude shifted in the same frequency range for 60 MHz between 100% and 20% isopropanol concentrations in water. Finally, given the large integration capability of novel microwave-microfluidic chips with other sensing technologies, a novel microwave-optical-fluidic platform could come closer to an ultimate cross-disciplinary system offering broad sensing and actuation versatility.
